# Human tracking robotic camera based on image processing for live streaming of conferences and seminars

**DOI:** 10.1016/j.heliyon.2023.e18547

**Published:** 2023-07-22

**Authors:** Atiq Ur Rehman, Yousuf Khan, Rana Umair Ahmed, Naqeeb Ullah, Muhammad Ali Butt

**Affiliations:** a Embedded Systems Research Group, Department of Electronic Engineering, Balochistan University of Information Technology, Engineering and Management Sciences, Quetta, 87300, Pakistan; b Department of Electrical Engineering, COMSATS University (Lahore) Campus, 53400, Punjab, Pakistan; c Institute of Microelectronics and Optoelectronics, Warsaw University of Technology, Koszykowa 75, 00-6625 Warszawa, Poland

**Keywords:** Robotic camera, RFID tracking, Image processing, Live streaming, Voila-jones algorithm

## Abstract

There are numerous scenarios where the photographer is in difficulty and unable to capture or shoot video as required. This could be due to several factors such as limited space, decreased visibility, and an obstacle in the way. Therefore, this project implements the idea to capture and shoot video of the desired subject through an automatically controlled robotic camera with no need for a photographic bloke. The system comprises functions such as detection, tracking, live streaming, and video/audio recording along with the features of Radio-Frequency-Identification (RFID). Therefore, this robotic camera will detect the desired subject, track and focus it with the help of its position driven through movable motors sensing the RFID tag in case the object is non-stationary. The video/audio will be recorded on a computer along with the live streaming available on an Android-based device. The Viola-Jones algorithm of the image processing technique is used to detect the particular subject features and C for accessing the movable camera protocols. The RFID transmitter and receiver are used to sense the RFID card and serve the purpose to track the subject using the algorithms of image processing, with the advantage of ignoring other obstacles between the camera and the detected subject. Thus, adding a novel functionality to the existing systems, that lacks the feature of focusing the camera on the subject, when an obstacle is detected in between. The live streaming is achieved wirelessly through an open-source platform X-operating system, Apache, MySQL, Php, Perl (XAMPP). The idea is verified through concluded arrangements in self-made scenarios in response to the speed, distance, light, and background noise of the detected subject, which delivered encouraging results. Therefore, the designed system can be used for live conferences, seminars, and other multimedia-required arrangements.

## 1 Introduction

Generally, for audio/video recording of conferences, seminars, and online lectures: there is always a need for external assistance in terms of a photographer. Congruently, there are scenarios, that make it very difficult to have the photographer each time. So, to make it easy and possible, this paper presents an idea of a robotic camera: which can focus on one person, and records the audio/video with the functions of live transmission via an Android-based app. Moreover, as, we have seen that the world is continuously moving towards modernization, individuals adopt new methods and ways of lifestyle in terms of roaming, traveling, attending functions, conferences, and different other activities with a keen to see what is happening around them and the world in particular. For this purpose, different systems are designed to capture and stream different aspects of their environment [1]. Similarly, live streaming of the news, conferences, online classes, photography, indoor and outdoor shooting have increased by a significant percentage [2]. Correspondingly, to the fast improvement in the silicon industry, many computer applications are designed to improve first-rate lives and live streaming dramatically [3].

One, of the noteworthy applications that enhance our lives is automation employing robotics [4]. A robot is a mechanical system/tool that performs automatic responsibilities, both in step with a direct human interface, pre-described programs, or fixed fashionable suggestions [5] or combined artificial intelligence techniques [6]. Therefore, the robots are meant to update people in monotonous, heavy, and risky tactics [7]. Nowadays, inspired by way of monetary motives, industrial robots are intensively being used in a variety of applications [8]. Similarly, regarding internal robotics autonomy, particular attention is given to cellular robots [9], due to their potential to discover their surroundings and non-alteration to at least one physical area. Therefore, we have seen the actual construction of versatile robots as presented in Ref. [10]. Moreover, the robotic industry is evolving in many new ways to upsurge the efficiency and accuracy of robotic systems [11]. The purpose is to reduce human effort and replace human duties with robotics [12].

Apart from controlling robotic systems through physical devices, different techniques and algorithms such as adaptive control, fuzzy logic, neural network, and image processing are used, which have proven beneficial to the field [13]. Specifically taking the algorithms of image processing, has shown diverse applications in the fields of medical [14], identification [15], security [16], and multimedia scenarios [17], thus adding unique characteristics to the functions of the robots. Recently, the face detection [18] method has become very popular. The fundamental purpose of face detection is to provide a more natural way to control and provide an intuitive form of interaction with the robotic system and enable the autonomous robot to detect objects using sensors or cameras to process this information into movements without remote control [19], real-time-activity and recognition from video frames in Ref. [20], and the recognition of the human group activity using Kerr Density Estimation (KDE) from video frames in Refs. [21,22]. As a result, the image processing technique has proven beneficial due to its better accuracy, speed, and less computational power requirements as reported in security systems [23], automobiles [24,25], automated parking lots [26], currency recognition [27], blood sample identification [28] and neural network-based hyperspectral image classification [29].

**Fig. 1:**
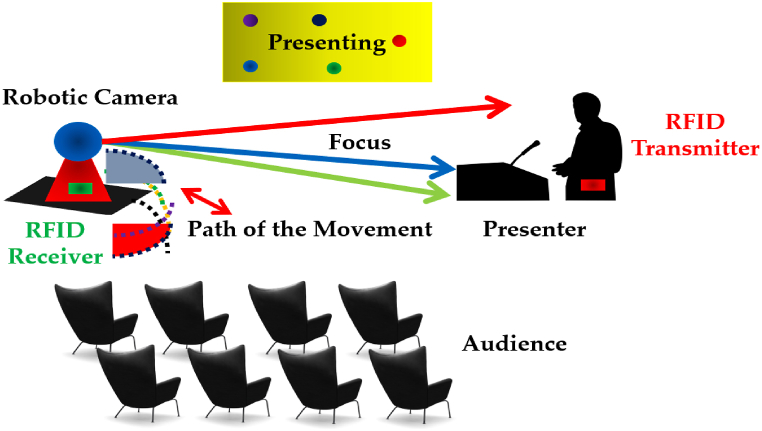
Proposed arrangement of the system to automize the live streaming using image processing, RFID tagging, and automated camera.

Similarly, RFID systems have shown profound advantages i.e., battery-free tags, low cost, and scalability in different fields of science, such as keyless-entry car systems [30], pedestrian navigation [31], Internet of Things (IoT) [32], healthcare systems [33], attendance-monitoring systems [34], and tracking analogies [35]. Combining these three different techniques i.e., autonomous robotics, image processing, and RFID system for functions such as recognition and tracking to live stream events such as conferences and seminars can be greatly automized as presented in this research work with Fig. 1, depicting the proposed arrangement.

Although, numeral systems are already designed for the purpose such as given in Ref. [36], using the concept to produce virtual environments for live streaming of conferences and seminars to address the issues of accessibility and sustainability for researchers and academicians. Similarly, another study is presented in Ref. [37], using face recognition property using the Viola-Jones algorithm for humanoid robots to detect different facial characteristics. The investigation as given in Ref. [38], uses a mini drone to recognize the face of the subject and track it accordingly. An idea is conceived in Ref. [23] to detect and track the subject within the area of a secure place using self-made scenarios i.e., speed, distance, and light intensity except for background noise. Similarly, another innovative approach is presented in Ref. [39] to track the subject by using the audio-visual device, where the subject/presenter is successfully tracked with the help of its sound while speaking by the imaging source without losing the focus. To capture different movements and activities of human beings i.e., handshakes, hug, kick, and many more both indoor and outdoor is studied in Ref. [40], using multiple feature algorithms and convolutional neural networks (CNN). Studying different motions of human beings is a difficult task in the sense to evaluate each position and structure as the number may end up in the billions. Therefore, a study investigated in Ref. [41], reviews multiple aspects and systems i.e., machine learning to achieve the prospects of human motion and detection in the best possible manner. A similar investigation is carried out in Ref. [42], using different methods for the detection of human motion such as Compressive tracking (CT), Multiple instance learning tracking (MIL), Fragment tracking (FRAG), and Tracking learning detection algorithm (TLD). The integration of the RFID in the systems used to track the motion of human beings is also of great interest, as given in Ref. [43], using the functionalities of the RFID tag in the devices named as ibraclet and iglove to detect and track different motions of the human during any activity. Correspondingly, tracking the motion of human beings and objects in their hands combinedly is studied in Ref. [44], using sequential Monte Carlo filtering and the RFID tag to avoid any uncertain circumstances in shopping malls and other customary places. Another benefitting use of the RFID tag is its integration with the Global Positioning System (GPS), to track the activities and movements of any human being or other objects as a whole in investigated in Ref. [45]. However, the usage of the RFID for general public is still prohibited due to concerns of security issues in some countries. To efficiently, detect and track the activities of human beings during conferences, an RFID tag system is introduced in Ref. [46], which uses the concepts of the reflection signals from an RFID tag to the reader antenna, and as a result, the activities of the human beings are detected, however, builds a complex scenario which is difficult to achieve in the required results of the system. Apart from these, some of the modern cameras have inner build capabilities to track the subject, when installed for a specific purpose.

However, these previously discussed systems are complex in design, unable to recognize facial characters when far away from the system and have the least focused camera and inconsistency to focus for a long time with needs of high-bandwidth and data-connectivity and prevailing battery issues. Similarly, will not provide tracking capabilities, if a certain feature is a little different from the one that is installed or given in the system. Correspondingly, the RFID tag used in these systems is for object detection in shopping malls and like places rather than human being tracking except GPS. Moreover, the cameras having built-in capabilities to track subject lacks the function to detect and track a specific subject of interest, instead track any subject which is within its premises. Apart from these, did not acknowledge the effects of background noise on the performance of the system, which is addressed in this research study.

Although, the device presented in Ref. [39] has a close resemblance with the ongoing research except that it uses the sound of the human as a tracking feature of the system, while in this research the RFID integration is used as a tracking feature providing higher efficiency of tracking of the subject without losing its sight. Whereas, in case of tracking through the sound of the subject, the sight and tracking capabilities of the system can be reduced.

Therefore, the robotic camera is fully automated in the proposed system with a simple design and can work without human guidance. The camera-carrying robot will detect the subject of interest with the help of the image-processing algorithm. Moreover, it will start tracking the speaker by controlling the camera's motion with the help of the installed servo motors both for vertical and horizontal movements with consistency [47]. Additionally, an RFID card is used to track and monitor the detected subject's movement [48]. Apart from the detection, the system will also serve the purpose of recording and live streaming on an Android-based application using HTTP-address for the purpose to be used on multiple devices simultaneously, requiring lower bandwidth and connectivity [49]. As an additional property of the system, one can save pre-images of the faces in the database to make it easy and speedy for the system in the detection process, while ignoring other faces during conferences and seminars thus saving battery life. Congruently, the images are saved with the name defined by the current date and time of the system at that instance, which will help in case if we require information about the specific events, whenever needed.

**Fig. 2:**
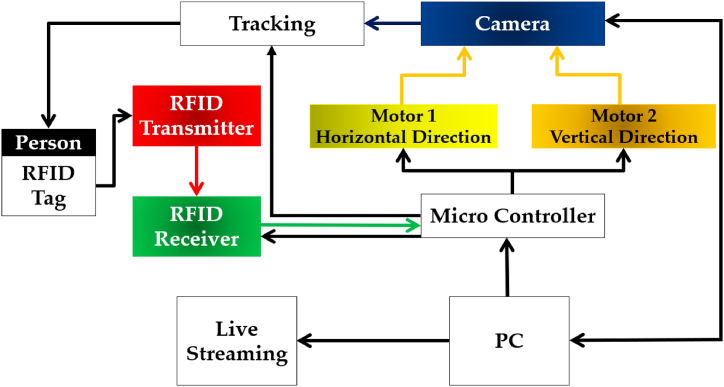
Systematic configuration of the proposed system.

A novel functionality in terms of designing a self-made RFID tag for the proposed system is achieved in this research work. This RFID tag will help in the detection of the subject, which is already detected by image processing. However, its addition serves a purpose as when the subject is detected by the image processing algorithm only, the focus of the camera can be lost when another object is detected between the robotic camera and the subject as in the case of systems comprising of the image processing algorithms and camera only. Therefore, the RFID in this case, when detected by the system with the desired subject simultaneously along the image processing algorithm, the robotic camera will retain its focus on the desired subject irrespective of the other objects detected between the two. Fig. 2, reflects the general configuration of the system.

The structure of this manuscript is as follows. Section 1 is devoted to providing the basic idea of the research and the recent works in the field. Section 2 details the basic methodology considered for the proposed system. Section 3 describes the particular implementation of different parts of the proposed system. Next, Sections 4, 5 present the self-made scenarios and analyzing of the results of the designed system respectively. Finally, Section 6 ends our study by highlighting the most relevant findings in our work.

## 2 Methodology

**Fig. 3:**
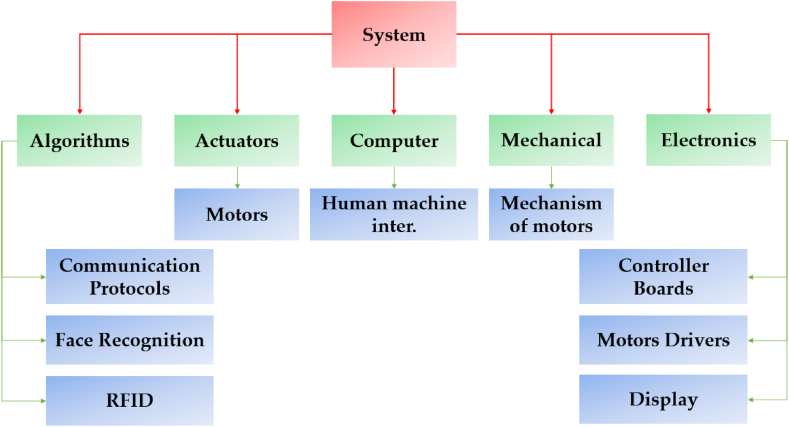
Overview of different parts of the designed system.

The working principle of the proposed system comprises image processing-based algorithms i.e., Viola-Jones for detection and tracking using an imaging source, the RFID tag for tracking, audio/video recording, and its live streaming. The overview of different parts of the system is depicted in Fig. 3.

Fig. 3, explains the different parts and connections of the system and is divided into six sections.

(a) Algorithms: This is the main part of the project associated with the programming of the system, i.e., detection and tracking with the complete working capability of the proposed system.
(b) Actuators: It comprises two servo motors to track the subject both in vertical and horizontal directions.
(c) Computer: Mainly responsible for the human-machine interface and live streaming.
(f) Mechanical: This portion of the project is responsible for the behavior of the servo motors at the center and the boundaries of the field of view (FOV).
(e) Electronics: Comprises a microcontroller board and necessary connections.

### 2.1 Viola-Jones algorithm

**Fig. 4:**
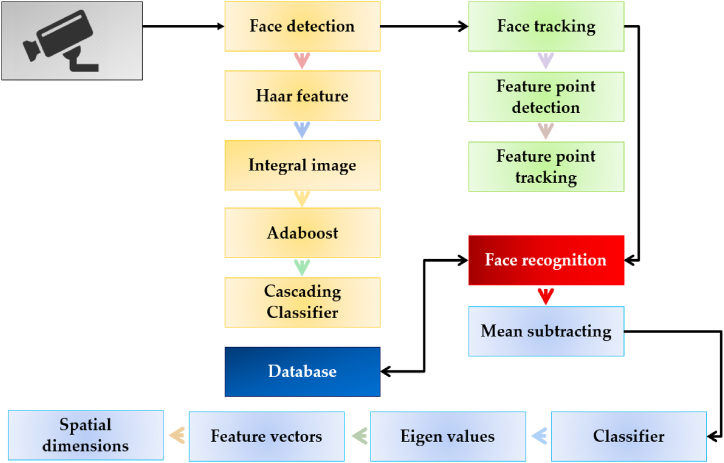
Flowchart of the Viola-Jones algorithm.

The image processing technique involves several algorithms depending on the system's requirements in terms of random clocks, computational power, capabilities, and need for time [50]. This research study is related to recognition, detection, and tracking functions by the system. Therefore, the algorithms related to detection and recognition procedures are mostly taken into account. Moreover, the techniques relating to these functions are numeral, which in turn have varying requirements in terms of resources depending on the structural parameters of the system [51]. However, the basic principle of all these techniques remains the same as to detect and recognize the subject. Therefore, in this paper, the image processing technique is used with Viola-Jones as the proposed algorithm to recognize and detect the subject, such as the facial features of the human being in this case. The basic principle of the process is to detect the object and segment the face (image) from a series of frames of the video. Later on, rescale the image to avoid any loss of information during the pre-processing stage. The flowchart of the algorithm is shown in Fig. 4.

The Viola-Jones algorithm is a framework proposed in Ref. [52], which intends to provide a solution for the quick processing of images with a high detection rate and the ability to run in real time. The major goal of the design is to provide an Integral Image for the detector which requires a lot of computing. Therefore, the Viola-Jones algorithm finds objects with good detection accuracy, while minimizing the computing time [53]. Moreover, it is more effective with the frontal images of the faces and can handle 45° rotation of the face in real-time with the capability for both vertical and horizontal directions. Therefore, comprises the following features.

#### 2.1.1 Haar features

**Fig. 5:**
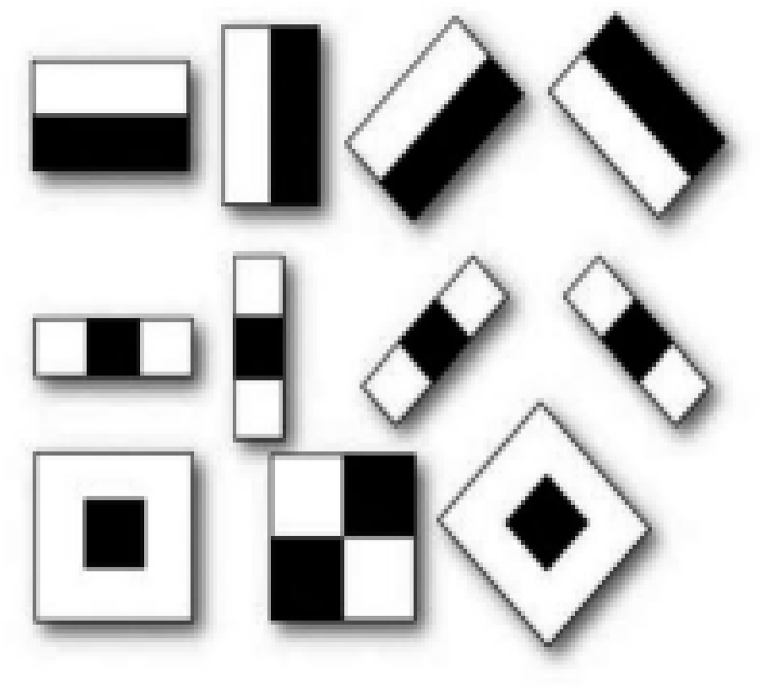
Two-rectangle aspects of the Haar feature [55].

The Haar feature gets the advantage of differences in the summed pixel values of the rectangular image area [54]. A rectangle with white and dark areas is used to calculate the number of pixel values in a digital image. Because it is an area divided into two equal parts, one dark and one white, this feature is known as the two-rectangle feature as shown in Fig. 5, imitating different types and sequences of the two-rectangle aspects.

The Haar characteristic is the function (F) that can be calculated by subtracting the feature in the dark areas F_Dark_ from the total values in the white areas F_White_ [56]. It can be stated mathematically by equation (1) as,

(1) $$F\;\left(Haar\right)=\sum F_{white}-\sum F_{Dark}$$

**Fig. 6:**
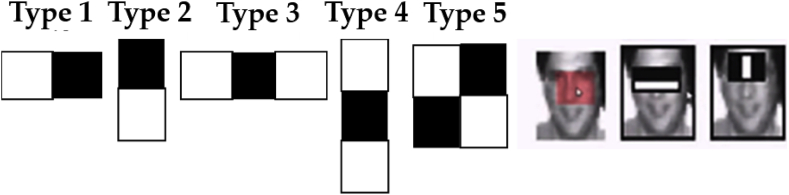
Image signifying the types of the Haar features.

Haar features and coefficients obtained from image convolution with positive and negative Haar functions enable us to detect such objects in the image as eyes, nose, and mouth. Moreover, we must train a computer to what a face is and what a non-face is [57]. Similarly, it can extract certain features and store them in a specific file. After getting a new image, this feature co-relates it with the previously saved file and matches the features. If they are matched, it will be recognized as the face otherwise as a non-face. Moreover, the Haar feature consists of 160,000+ features, with Fig. 6, signifying some of them, with the dark region replaced by +1 and the white region by −1. By applying the ‘type 2’ algorithm for eye detection and ‘type 3’ for nose detection on the image, it subtracts the pixel's value under the white region from the pixel's value under the dark region and generates an output containing a single value [57].

#### 2.1.2 Integral image

**Fig. 7:**
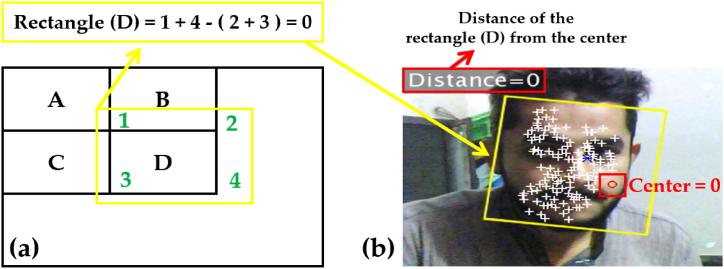
(a) Rectangle solution in Viola-Jones (b) Real-time simulation for rectangle detection.

This feature concludes the sum of the pixels within the rectangular box as shown in Fig. 7(a). An integral image is used to calculate the sum of all pixels inside the rectangular box using only the four values at the corners of the rectangle [58]. Referencing Fig. 7**(**a): the process is carried out to calculate the value of the rectangle D as depicted by a solid (yellow) rectangle. Moreover, by adding the pixel value of corresponding diagonal corners and subtracting both diagonals from each other to calculate the pixels of image corners and then using an integral image property by summing them. Equation (2) reflects the calculated equation for the determination of the rectangle (D) in this research work.

(2) *D = A* + *(A + B + C + D) – (A + C + A + B)*

The symbol ‘D’ is the rectangle of interest in this research work and all the calculations relating to summation and detection by the system are achieved through it. Therefore, equation (2) allows us to produce a rectangle along the areas that are selected by the Viola-Jones algorithm corresponding to different feature points and regions of the face. Similarly, ‘A, B, and C are respective regions (values) around the rectangle (D), helping in the overall calculation and determination of the rectangle (D) as shown in Fig. 7(b). Moreover, the Distance in Fig. 7(b), is the separation of the rectangle (D) from the center of the screen as shown by a red (hollow) circle. It acts as the pre-defined coordinates for the motors of the camera to allow tracking of the subject's face discussed in the upcoming sections.

Thus, a frame is detected from the video and a rectangle is inserted around it as imitated in Fig. 7(b). The Integral image rapidly calculates the summations of the sub-regions of the image: which facilitate the summation of pixels and can be performed in constant time with the processed image converted into greyscale initially.

#### 2.1.3 Adaboost

**Fig. 8:**
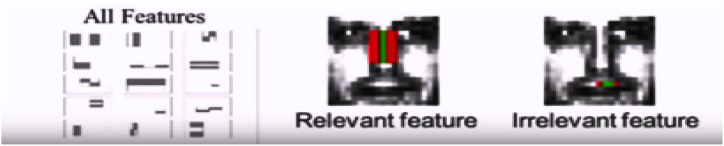
Eigen features detection in an image.

As stated previously, there can be approximately 160,000+ features within a detector that needs to be calculated. However, it is to be understood that only a few sets of features will be helpful among all these features to identify a face such as the eigen feature points as shown in Fig. 8 [59].

In Fig. 8, the detection of the relevant features is shown where the bridge of the nose is yielding the highest values at this position of the image. On the other side, the image does not give the relevant information because the region at the upper lip is constantly producing no evaluation. Therefore, the relevance and irrelevance can be easily identified by using the property of the Adaboost.

#### 2.1.4 Cascading classifier

**Fig. 9:**
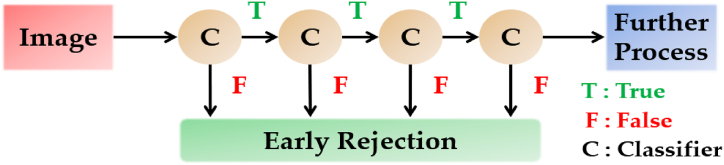
Block diagram of the Cascade classifier.

The cascade classifier is designed to speed up the face detection procedure while ensuring accuracy. It is divided into multiple steps, each of which is utilized to complete a thorough categorization by grouping all of the available features and framing the items in the image to detect the faces [60]. The block diagram of the cascade classifier is illustrated in Fig. 9.

The primary principle of the Viola-Jones face detection algorithm is to test the detector, usually via similar pictures (whenever with a brand-new length). Although an image is thought to include one or more faces, thus making it evident that a significant quantity of the evaluated sub-home windows from this image would still be negative i.e., non-faces. So, the rule is to give attention to discarding non-faces quickly and spending extra time on all likelihood face regions. Thus, the cascade classifier is used, which comprises tiers: each containing a robust classifier [61]. All the stages are grouped into several stages, wherein every stage has a particular variety of capabilities. The activity of every degree is used to decide whether a given sub-window can be a face or not. Hence, a given sub-window is discarded as no longer a face, if it fails to any degree. This is the process behind the detection of the subject using the imaging source in the following sequence i.e., (a) getting frames of the video (b) converting it to greyscale (c) integrating it into the rectangle.

**Fig. 10:**
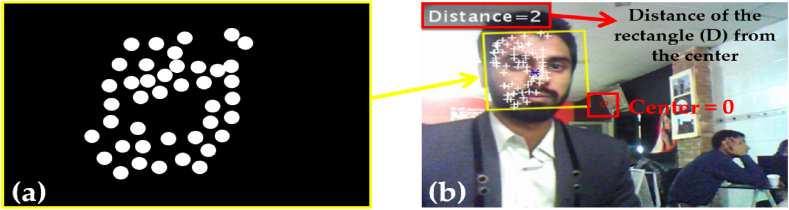
(a) Features detection on the face. (b) Face detection by real-time simulation of the system.

Therefore, it will detect the Haar features of that image and save it in the form of a 2D matrix. From the grey image, it will detect the eigen feature points and replace the old points of the image with a new frame from the camera by converting the rectangle represented by [x, y, w, h] into a matrix form of [x, y] coordinates of the four corners. Thus, it transforms the bounding box to display the orientation of the face. The rectangle features and detection points are shown in Fig. 10(a) and (b), from the real-time simulation of the system.

### 2.2 Motion control

**Fig. 11:**
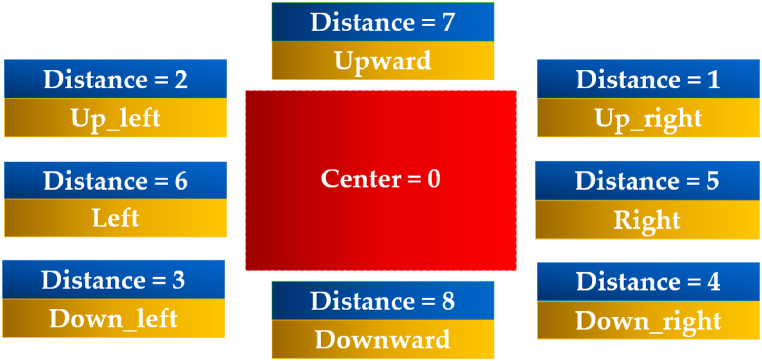
Coordinates of the rectangle to control the motion of the motors.

The Viola-Jones algorithm is used to detect the object and correlation is used for the recognition property. Moreover, the next step is to control the movement of the camera for the detected and recognized face. As, the motion of the camera is controlled by two servo motors, i.e., (a) horizontal movement (Tilting the camera) and (b) vertical movement (Panning the camera) driven by an Arduino microcontroller. Moreover, there are eight pre-defined coordinates in which the imaging source will follow the subject's movement using the detected rectangle (D) and its distance from the center of the screen, as imitated in Fig. 11.

**Fig. 12:**
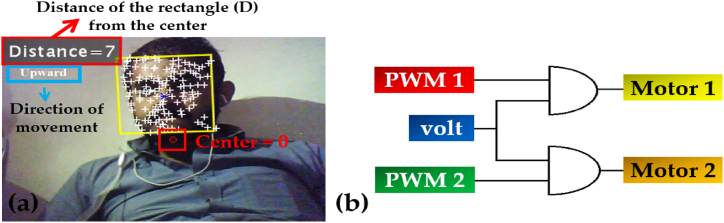
(a) Subject and distance detection by the camera. (b) Gate circuitry to drive the motors.

Here ‘Distance’ is the distance, which will locate the detected subject and move towards that position to follow it; at the center i.e., center = 0, the camera will retain this position in case of no detection. However, after the subject has been detected, it will move by this center point as a reference to efficiently track the subject. Fig. 12(a) depicts the detection of the face by real-time simulation of the system and its distance from the center, with Fig. 12(b) imitating the connections used for the servo motors.

### 2.3 RFID interfacing

RFID is used for tracking after the face of the subject is detected. Moreover, comprises a receiver interfaced with the Arduino microcontroller and a transmitter with the desired subject. The RFID tag is a transponder with a coil and external antenna supporting a specific frequency. The RFID used in this project, is passive, having a frequency of 125 kHz. Thus, when this frequency of the tag is matched with the receiver's coil, the card will be detected. The procedure follows when the camera detects the face of the subject. Two cases will be taken into consideration by the system (a) when the RFID is detected by the system, the process of detection and tracking will occur concurrently of the desired subject, therefore ignoring other objects in between the camera and subject (b) when the RFID is not detected, the process of the detection and tracking will be considered without the property of ignoring other objects in between.

### 2.4 Audio and video recording

The designed robotic camera has the added functionality of recording the audio/video files of the ongoing conference or seminar. The recording of the audio/video begins at the time when the desired subject is detected both by the camera and the RFID tag. Moreover, provides the functions to save and record the audio/video in two separate directories, which when needed can be opened and seen or listened. The audio file of the recording is saved in the ‘‘wav’ format, while for video recording the frames are saved and cascaded in the end to form a video in ‘avi’ format. Additionally, the names of the files of the audio/video recordings are saved with the day and time of the respective event held, providing easiness in finding the files of a concerned event.

### 2.5 Live streaming

The Html language is used, for the design of a web page to get the video frame-by-frame for live streaming. For this purpose, the IP address of the host computer is achieved with the help of open-source Xampp software [62]. Therefore, a hotspot of an Android phone is needed to maintain a connection with the host computer for the working of the software. For streaming on multiple platforms, the devices need to have an active connection of data and the IP address of the host computer.

## 3 Implementation

**Fig. 13:**
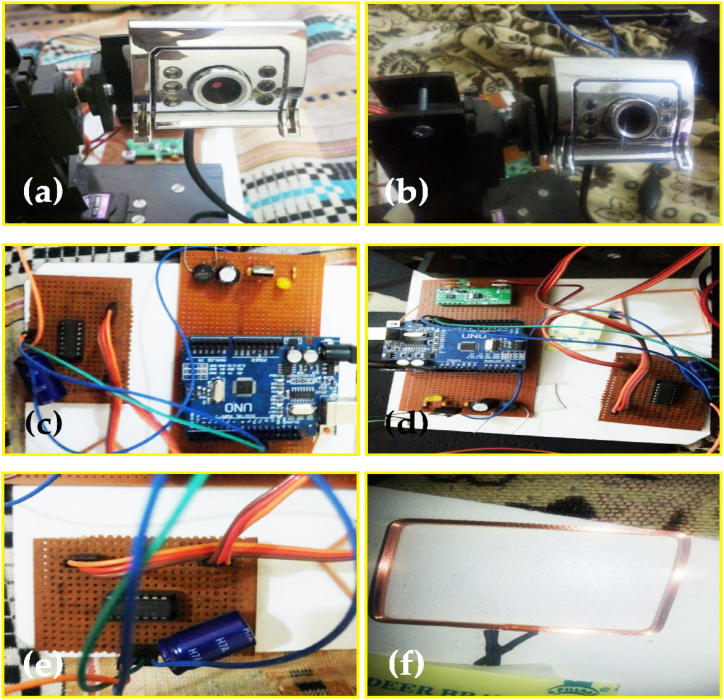
The stepwise implementation of the system (a) Interfacing of the camera with PC (b) Connecting motors with a camera (c) Connecting motors with micro-controller (d) Connection of micro-controller (e) Connection of motors (f) RFID coil.

This section comprises of implementation of different parts of the system as shown in Fig. 13(a–f). Therefore, Fig. 13(a) imitate the connection of the camera with the PC. Fig. 13(b) shows the connections of the servo motors with the camera for its horizontal and vertical movement. Fig. 13(c) reflects the connections of both of the servo motors with the microcontroller. Fig. 13(d) reports the microcontroller and its connections. Fig. 13(e), investigates the connection of the camera and micro-controller to the PC. While Fig. 13(f) displays the coil of the RFID tag.

The stepwise, arrangement of different components of the system is as follows.

(a) Several hardware components, which are programmed i.e., starting from the selection of the micro-controller, the Arduino Uno is used in this system, which is programmed using the Arduino (IDE) for the purpose to drive the servo motors (For vertical and horizontal directions). Moreover, comprises the RFID receiver which is programmed to receive the signals from the RFID transmitter. Similarly, the programming mechanism for wireless communication is also performed in this step. The basic properties of the Arduino Uno and RFID tag are given in Table 1 and Table 2, respectively.

**Table 1:** Specifications of the Arduino Uno micro-controller.

Microcontroller	Atmega328 P
Operating voltage	5 V
Input voltage	7–12 V
Digital I/O pins	14 (6 provides PWM signal)
Analog input pins	6
Clock speed	16 MHz
length	68.6 mm
Width	53.4 mm
Weight	25 g

**Table 2:** Specifications of RFID tag (RDM6300).

RFID tag	RDM6300
Frequency	125 kHz
Baud rate	9600
Interface	ITL RS232
Voltage	5 V
Current	<5 mA
Receiver range	20∼50 mm
Winding size	46.0 mm × 32 mm × 3 mm
Module size	38.5 mm × 19 mm × 9 mm
(b) The second step comprises programming the software components of the system i.e., Viola-Jones algorithms for the detection of the facial features and tracking of the desired subject using MatLab.
(c) Due to the ban on the RFID tag in the country, the RFID tag for this system is designed and built from scratch with distinctive and required properties for the system.
(d) Similarly, for a full-pledged system, the software and hardware components i.e., microcontroller, camera, RFID tag, servo motors, XAMPP, and PC are connected to get the desired outputs of this research study.

## 4 Illustration of the room for self-made scenario's

**Fig. 14:**
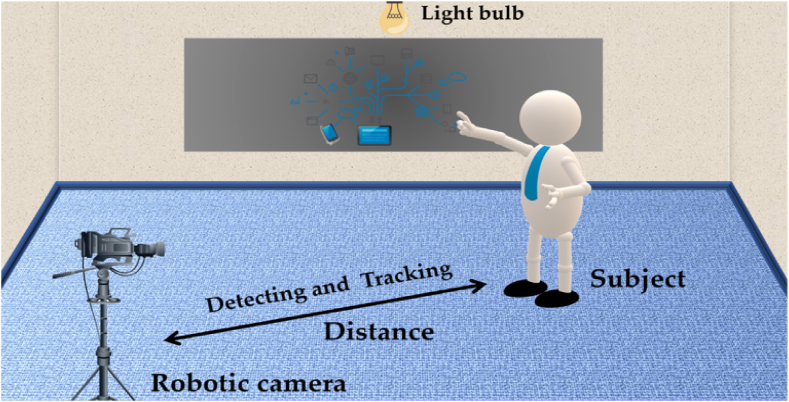
Image depicting the illustration of the room for testing the system in self-made scenarios.

To analyze the working performance and testing of the system in self-made scenarios, a room as shown in Fig. 14, is used, where all the necessary arrangements were made. The room comprised 4 × 6 × 4.5 (length × width × height) meter (m) dimensions and was completely emptied. Therefore, the self-made scenarios were designed as such, to present the complete picture of the system in terms of its detection and tracking capabilities. Therefore, the detection is the first property of the system, where the facial features of the subject are determined when entered into the premises of the imaging source i.e., the camera requires less computational resources and the system's speed for operation. While, tracking is the second property of the system, which is initiated after the detection process by the system. Moreover, is associated with tracking the movements of the detected subject using the imaging source and the RFID tag. Therefore, requires high computational resources and power, because of three concurrent processes occurring at the same time i.e., (a) detection (b) tracking using servo motors, and (c) generation of signal for the RFID tag. Therefore, the below-mentioned self-made scenarios (Section 5) are investigated for these two properties i.e., detection and tracking, and calculations were performed for the system in the room as shown in Fig. 14. To make it understandable, let's take an example of the minimum and the maximum distance of the subject that is detectable and trackable by the robotic camera i.e., 2–10 feet roughly representing 0.6m–3m (distance in meters) at most. Therefore, this distance i.e., 0–10 feet (0–3 *m*) is divided into five equal imaginary points located at 2, 4, 6, 8, and 10 feet from the robotic camera, to investigate the performance of the system, where each point is representing a decrease of about 20% in the system performance respectively. Although the system uses the System International (SI) units system, however for easiness to understand the behavior of the system, distance is elaborated in feet as well. Therefore, the normal functioning of the designed system is described in feet i.e., 0–10 feet also, equalling to 0–3 *m*. So during the calculation process, every 2 feet i. e, 0.61 *m* represents about 20% efficiency (working of the system both in terms of detection and tracking), with the highest efficiency i.e., 90–95% achieved when the distance between the system and subject is minimum i. e, 2 feet (0.61 *m*) and lowest efficiency i.e, 5–10%, when distance is 10 feet (3.05 *m*).

## 5 Results and discussion

**Fig. 15:**
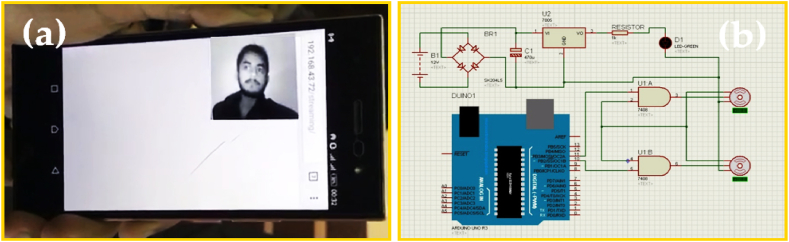
Results of the system obtained after running (a) Live streaming (b) connection of servo motors.

**Fig. 16:**
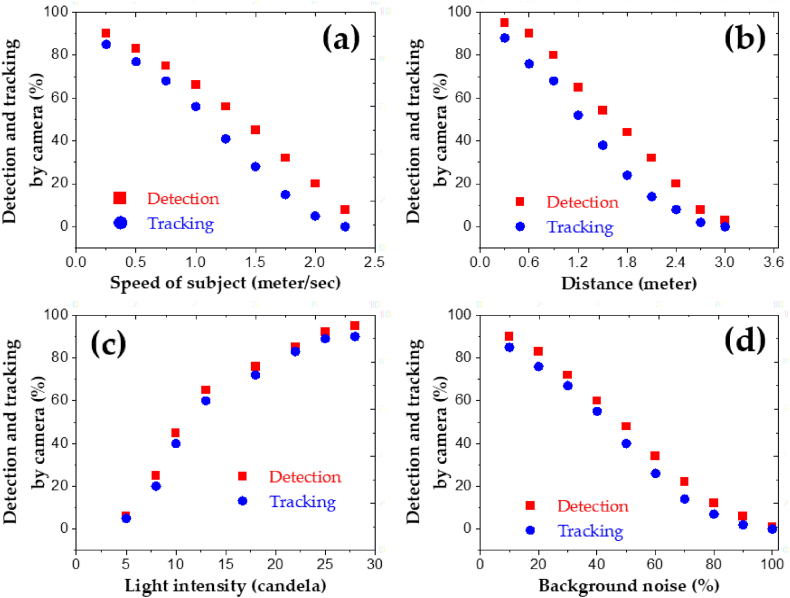
Output graphs presenting detection and tracking properties of the designed system (a) Speed. (b) Distance. (c) Light intensity. (d) Background noise.

The results of the system obtained after its running are shown in Fig. 15. Where Fig. 15(a), reflects the live streaming capability of the system with Fig. 15(b) imitating the connections and design of the whole system in the proteus software. Similarly, Fig. 16, reflects the quantitative evaluation of the system results in terms of detecting and tracking obtained after its running as reported in Fig. 16(a) vs speed, 16(b) vs distance 16(c) vs light intensity, and 16(d) vs background noise.

### 5.1 Speed effects

The speed will affect both qualities of the system, i.e., detection (facial features of the subject) and tracking (movements of the subject). The arrangements corresponding to the speed of the subject were implemented on that side of the room that was 4 *m* long (length). The wall was made clear of the drawings and a portion in the middle was left for presentation in the form of a large poster. The robotic camera was made focused on the subject. The performance of the system in terms of detecting and tracking was calculated while maintaining a distance of 1 *m* between the subject and the robotic camera. Therefore, as the average speed of the human being, is from 0.7 m/s – 1.2 m/s, the system performed well as the subject moved alongside the poster. However, as the speed of the subject increased, i.e., moving alongside the poster and picking up the markers or water, the capability of the system decreased gradually.

For this purpose, two approaches were taken the length Infront of the poster was calculated i.e., 3 *m*, and the time taken to cover this distance by the subject while moving back and forth with normal speed through a stopwatch. Gradually, the speed of the subject was increased and several values were calculated and an average was taken for calculation of the response of the system.

The second approach was the calculation of the speed through a mobile device. After taking several calculations, the average value was taken and drawn against the properties of the system. Moreover, it was noted that the tracking of the subject is more vulnerable to speed as compared to the detection property producing an inverse relationship as shown in Fig. 16(a).

### 5.2 Detection, tracking vs. distance

Detection of a subject by the imaging source using the image processing technique is the central part of the system. However, distance limitation is present, as the system will detect and track the subject when the distance between them is in the range of 0.3 *m*–3 *m* i.e., roughly 2–10 feet. The arrangement was self-made in the room facing the 4 *m* wall with a poster in the middle and the robotic camera was implemented at a distance of 0.3 *m* from the subject and was moved gradually away up to 3 *m*. Thus, the results were concluded and are shown in Fig. 16(b), presenting an inverse relationship.

### 5.3 Lighting effects

Light is another aspect of the system's correct operation in terms of detection and tracking of the subject via the camera. To calculate the system's response, a poster is implemented in the middle of the 4 *m* facing wall and the subject is ready to deliver it. Therefore: a light bulb corresponding to 5 W (roughly 5–6 cd) power is installed above the poster, where its power is gradually increased up to 30 W (30–33 cd). During this time, the capability of the system is noted through successful detection of the subject up to 90% and tracking up to 85% while maintaining a distance of 1 *m* between the robotic camera and the subject. However, the tracking function of the system concerning light is more dependable as compared to the detection by the system, with the following observations achieved using different light intensities, as shown in Fig. 16(c).

### 5.4 Background noise

Background noise is also an essential factor that decides the system's working both in terms of successful detection and tracking of the subject. Moreover, the system is designed basically for indoor use instead of outdoor. Thus, indoor background noise can be in the form of reduced light intensity, crowded places or backgrounds, and invisibility. The approach selected to acknowledge the effects of the background noise, the size of the poster which was initially 3 *m* long (in the middle of the 4 *m* long wall), was reduced to 2 *m*, allowing a blank space of 0.9 *m* on each side of the poster, while, maintaining a distance of 1 *m* between the robotic camera and the subject. Thus, for calculation purposes, the sides of this poster were gradually covered with other colorful images (initially white), which in turn decreased the visibility of the subject and the capability of the system in terms of detecting and tracking. Therefore, the following calculations were noted, presenting an inverse relationship between the background noise and the working of the system as imitated in Fig. 16(d). As an acknowledgeable fact, background noise can be suppressed by using higher intensities of light, clearer background, and minimum distance between the robotic camera and the subject.

### 5.5 Limitations

During the designing of the system, two types of limitations i.e., software and hardware of the system were taken into account. The software portion was related to the Viola-Jones algorithm to efficiently detect and track the subject. Therefore, during the self-made scenarios, they were noted and presented as the limitations of the system which are given below.

• Is not effective in detecting tilted or turned faces efficiently
• Sensitive to lighting conditions by using different intensities of light
• Sub-windows overlapping for the same face
• Cannot detect multiple subjects at once as it is designed for a single user

Similarly, the limitations related to the designing of the system are.

• Short-range RFID tag as it was built from scratch due to a ban in the country for the general public
• Low-power servo-motors due to lower power consumption and usage of lower-end systems
• Lower-computation resources for cost-effectiveness

## 6 Conclusions

Implementation of a robotic camera using the Viola-Jones algorithm of the image processing technique for recording and live streaming of conferences and seminars is an addition to the existing models that cannot fix the focus of the camera on the subject when an obstacle is detected in between. Therefore, this system provides the solution in terms of using the Viola-Jones algorithm to detect and save the face of the presenter within the database of the computer. Moreover, the system will fix the focus of the camera on the detected subject and will track it according to the movements of the subject. However, if any object is detected between the camera and the desired subject, the focus of the camera can be lost. Therefore, the RFID tag is an innovative idea added to the system. Therefore, when it is detected by the system, the system saves the face of the subject against this RFID tag holding it at the movement. This will allow the camera to focus on the subject's face, movements and track them accordingly. Moreover, provides uniqueness i.e., if any obstacle is detected between the camera and the detected subject, the focus of the camera will remain on the subject, without losing its site. Live streaming is another function added to the system for a wide range of coverage and communication. Apart, from these, the audio/video recording of the conferences and seminars are also saved, with the name given by the respective time and day of the event for easy access and live streaming in the future if required. Moreover, the model is tested in self-made scenarios to test its detecting and tracking capabilities, which performed exceptionally well. Therefore, this model can detect and track the subject with 80%–90% accuracy with an average speed of human movement and up to 2 *m* of distance. The designed system performs well in well-lighted conditions and performance is limited in scenarios with dim light and a high percentage of background noise in terms of darkness, random colors, or smokey conditions. In future work, the performance of the system can be increased by using long-range RFID cards, high computing power and resources, high-power servo motors, multiple wireless cameras, and global live streaming. The model is well suited for application areas like live streaming, online services, and videography.

## Author contribution statement

Atiq Ur Rehman: Conceived and designed the experiments; Performed the experiments; Wrote the paper.

Yousuf Khan: Contributed reagents, materials, analysis tools or data; Wrote the paper.

Rana Umair Ahmed: Performed the experiments; Wrote the paper.

Naqeeb Ullah: Conceived and designed the experiments; Performed the experiments.

Muhammad Ali Butt: Analyzed and interpreted the data.

## Data availability statement

Data will be made available on request.

## Additional information

No additional information is available for this paper.

## Declaration of competing interest

The authors declare that they have no known competing financial interests or personal relationships that could have appeared to influence the work reported in this paper.
